# Human papillomavirus vaccine-associated premature ovarian insufficiency and related adverse events: data mining of Vaccine Adverse Event Reporting System

**DOI:** 10.1038/s41598-020-67668-1

**Published:** 2020-07-01

**Authors:** Li Gong, Huan-huan Ji, Xue-wen Tang, Ling-yun Pan, Xiao Chen, Yun-tao Jia

**Affiliations:** 1 Department of Pharmacy, Ministry of Education Key Laboratory of Child Development and Disorders, National Clinical Research Center for Child Health and Disorders, China International Science and Technology Cooperation Base of Child Development and Critical Disorders, Children’s Hospital of Chongqing Medical University, Chongqing Key Laboratory of Pediatrics, Chongqing, 400014 China; 2 School of Pharmacy, Chongqing Medical University, Chongqing, 400016 China; 3 TAIMEI Techenology, Zhejing, 314031 China; 4 Chongqing Three Gorges Central Hospital, Chongqing, 404000 China; 5 Department of Pharmacy, Chongqing Emergency Medical Center, Chongqing University Central Hospital, Chongqing, 400014 China

**Keywords:** Risk factors, Public health, Epidemiology

## Abstract

We detected disproportionate reports of premature ovarian insufficiency (POI) and related events, including amenorrhea, menstruation irregular, FSH increased, and premature menopause, following human papillomavirus (HPV) vaccine from FDA Vaccine Adverse Event Reporting System (VAERS). The signal was detected by the methods of Bayesian Confidence Propagation Neural Network (BCPNN) and Multi-item Gamma Poisson Shrinker (MGPS). When both methods detected a positive result, a signal was generated. Besides, time-scan map is drawn based on the IC value and 95%CI of BCPNN, if the IC curve showed a steady upward trend and the 95%CI narrowed, the signal was stable and strong association.The results showed that there were not POI reports of HPV vaccine, but VAERS received a total of 2, 389, 27 POI related events for HPV2, HPV4, HPV9 respectively from the year of marketed to 2018. No signal was detected for HPV2. HPV4-POI ralated events were all detected as signals by two methods. There was only one signal of menstruation irregular for HPV9. Time scan of HPV4-POI ralated events showed those signals were stability and strong association, but not for HPV9. Our results only represent statistical association between HPV vaccine and POI related events, causal relationship needs further investigation.

## Introduction

Human papillomavirus (HPV) is the most common viral infection of the reproductive tract and is the cause of a range of conditions in both men and women and cervical cancer is caused by certain types of HPV. It was estimated that 569,847 new cervical cancer in 2018, of which 311,365 deaths around the world^1^. Continuous infection of high-risk HPV types is highly associated with the development of cervical cancer, and administration HPV vaccine can effectively prevent cervical cancer. Three prophylactic HPV vaccines are currently available worldwide, including HPV bivalent recombinant vaccine (HPV2), HPV quadrivalent recombinant vaccine (HPV4), HPV 9-valent recombinant vaccine (HPV9).

Premature ovarian insufficiency (POI), also known as premature menopause or premature ovarian failure^2^, is a clinical syndrome defined by loss of ovarian activity before the age of 40, which is characterized by menstrual disturbance (amenorrhea or oligomenorrhea) with raised gonadotrophins and low estradiol ^3^. Women with POI have a little chance of spontaneous pregnancy, and no interventions could increase ovarian activity and natural conception rates ^3^. With the widespread of HPV vaccine, there were an increasing number of reports and studies on HPV vaccine-POI combination. For example, case reports and case series study reported a possible link about adolescent POI following HPV4 ^4^, but a population-based retrospective cohort study found no elevated risk of POI after HPV4 administration ^5^. Although these observations were not identified the possible risk for HPV vaccine-POI association, we connot exclude HPV vaccination has a role in pathogenesis of POI ^6^. So far, there are no reports and studies of HPV2 and HPV9-POI combination, and whether various HPV vaccines associated with POI is still inconclusive.

Published adverse reactions about HPV vaccines were mainly available form clinical trials, which might not reflect the full safety characteristics of HPV vaccines because of strict trial design, relatively small sample size and short duration of follow-up. Large-scale adverse event spontaneous reporting system is an important data source for identifying and discovering new or rare adverse reactions and after marketing. Signal detection from spontaneous reporting system (SRS) is carried out by many regulatorty authorities, such as the US Food and Drug Administration (FDA) ^7^, the World Health Organization Uppsala Montoring Centre (WHO-UMC) ^8^, the Netherlands Pharmacovigilance Centre Lareb. ^9^, to evaluate possible association between vaccine/drug and event combination, which including detection of novel signal, strengthening or weaking of existing signal and providing evidence for or against exicting safety concern not evaluated by data mining.

Published researches have supported or opposed the possible association between HPV4 vaccination and POI. We designed this study to evaluate the statistical association and detect the signal of POI and related events of three HPV vaccines, and to provide evidence for or against exicting HPV4-associated POI concern based on the data mining and signal detection method. The research data was obtained from the Vaccine Adverse Event Reporting System (VAERS).

## Results

### Descriptive analysis

**Figure 1:**
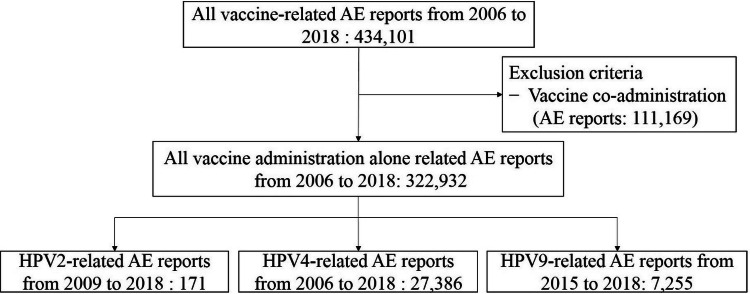
Flow diagram of case inclusion in this study.

Between 2009 and 2018, VAERS received a total of 275,595 vaccinated alone reports, including 171 for HPV2; from 2006 to 2018, there were 322,932 single use reports, of which 27,386 were HPV4; 143,909 were extracted from 2015 to 2018 and 7,255 for HPV9 (Fig. 1). In the adverse event reports of each HPV vaccinated alone, reports of amenorrhea, irregular menstruation, FSH increased, and premature menopause were extracted, and there were 2 reports for HPV2, 389 for HPV4, and 27 for HPV9.

For the types of the reporter, it was mainly come from healthcare provider (HPV2 accounting for 100.00%, HPV4 for 54.76%, HPV9 for 29.63%), suggesting that the report was authentic and professional. The healthcare providers include physician, physician assistant, medical assistant, certified medical assistant, nurse, registered nurse, licensed practical nurse, nurse practitioner, health professional, healthcare worker, doctor of pharmacy, pharmacist, pharmacy technician, physical therapist.

The age distribution was mainly concentrated on 9–18 years old (HPV2 accounting for 100.00%, HPV4 for 50.64%, HPV9 for 55.56%), and the proportion of less than 40 years old is 100.00%, 77.12%, and 70.37%, respectively for HPV2, HPV4 and HPV9. For serious adverse event (SAE), there are 0, 55 and 1 reports for HPV2, HPV4 and HPV9 respectively. The general characteristics and composition of each HPV adverse event reports are shown in Table 1.

**Table 1:** General characteristics and composition of adverse event reports.

	HPV2	HPV4	HPV9
Total reports	2	389	27
Type of reports			
Healthcare provider	2(100.00%)	213(54.76%)	8(29.63%)
Manufacturer	/	34(8.74%)	1(3.70%)
Patient/parent	/	34(8.74%)	4(14.81%)
Other/unknown	/	108(27.76%)	14(51.85%)
Age groups (years)			
< 9	/	1(0.26%)	0
9–18	2(100.00%)	197(50.64%)	15(55.56%)
19–26	/	97(24.94%)	4(14.81%)
27–40	/	5(1.29%)	0
> 40	/	1(0.26%)	0
Unknown serious adverse event	/	88(22.62%)	8(29.63%)
Death	/	1(0.26%)	0
Life-threatening	/	11(2.83%)	0
Hospitalized	/	14(3.60%)	0
Prolonged hospitalization	/	4(1.03%)	0
Disability	/	25(6.43%)	1(3.70%)

### Signal detection

Signal detection was performed on POI related events of three HPVs. The results are shown in Table 2. The number of HPV2 reports did not reach the test standard, and no signal was detected. HPV4-associated amenorrhea, menstruation irregular, FSH increased, premature menopause were positive results by two methods, suggesting statistically significant elevated risk of HPV4-associated POI and related events. For HPV9, irregular menstruation was detected as signal, but the other 3 adverse events did not detect positive result by MGPS.

**Table 2:** The results of signal detection for POI related events.

Events	Amenorrhea			Menstruation irregular			FSH increased			Premature menopause		
HPV	N	BCPNN (IC-2SD)	MGPS (EB05)	N	BCPNN (IC-2SD)	MGPS (EB05)	N	BCPNN (IC-2SD)	MGPS (EB05)	N	BCPNN (IC-2SD)	MGPS (EB05)
HPV2	2	–	–	0	–	–	0	–	–	0	–	–
HPV4	138	2.95	8.80	239	2.97	6.92	6	0.82	7.17	33	2.30	6.90
HPV9	8	0.56	1.96	15	1.47	2.55	1	–	–	4	− 0.04	1.55

### Diagram of time scan

**Figure 2:**
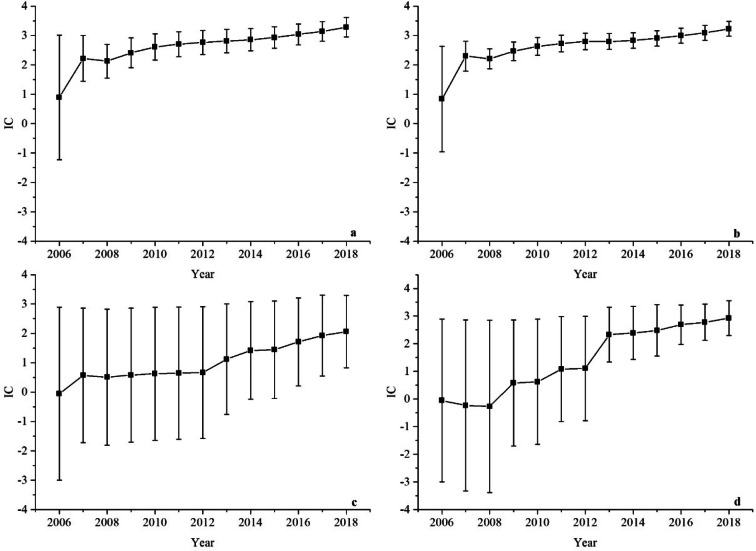
Time scans of IC for HPV4-associated POI raleted events from the year 2006 to 2018: (**a**) HPV4-amenorrhea association, (**b**) HPV4-menstruation irregular association, (**c**) HPV4-FSH increased association, (**d**) HPV4-premature menopause association.

**Figure 3:**
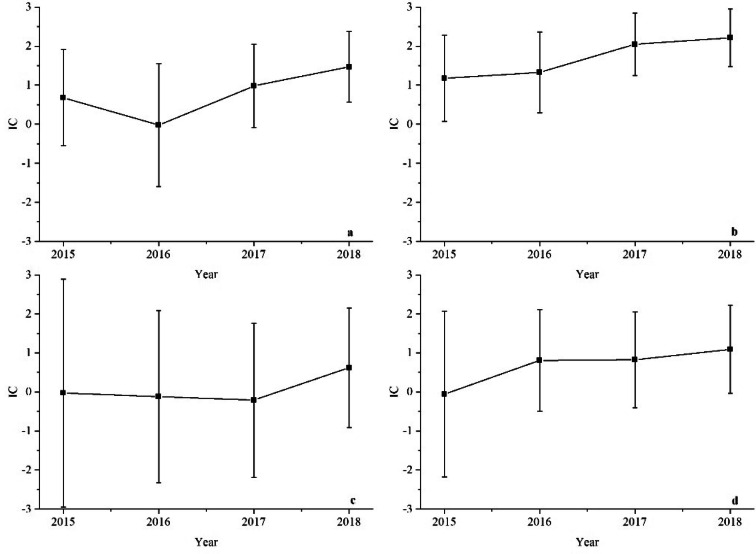
Time scans of IC for HPV9-associated POI related events from the year 2015 to 2018: (**a**) HPV9-amenorrhea association, (**b**) HPV9-menstruation irregular association, (**c**) HPV9-FSH increased association, (**d**) HPV9-premature menopause association.

A time-scan map is drawn for the HPV-associated POI related events to show the change tendency of the signal over time. When the lower 95% confidence interval (CI) limit of the IC for the HPV vacccine-AE combinations changes from a negative a positive value, the signal appears. When the curve of IC shows a steady upward trend and the 95%CI narrowed, it indicates that the signal tends to be stable and strong association ^10^. The results are shown in Figs. 2–3, of which Fig. 2 show the HPV4- associated POI related events and Fig. 3 for HPV9.

The time scan of HPV4-associated amenorrhea and menstruation irregular shown in Fig. 2a and b, which have a similar feature. From the diagrams in Fig. 2a and b, we can see the value of IC increase markedly and the lower 95%CI limit above zero in 2007 as the number of reports of HPV4-associated POI related events increasing, which indicates the two signal first appeared in 2007 and earlier than some case reports and researches. From 2007 to 2018, the interval of the IC becomes smaller, which means a stable signal and strong association.

Figure 2c shows the time scan of HPV4-associated FSH increased. The lower 95%CI limit always below zero, because few reports are received in VAERS from 2006 to 2015. As the reports increasing, this makes the value of IC increasing and the lower 95%CI limit above zero in 2016, which means the signal first generated in 2016. From 2016 to 2018, the bars around the IC are smaller, which shows the signal is stable and a strong statistical association.

For the HPV4-associated premature menopause (Fig. 2d), because no reports are accepted about this association, the IC curve decreases from 2006 to 2008, apart from a slight increasing after 2009 when the adverse event was reported. In 2013, there are 11 reports received of this event, then the IC increasing rapidly and the lower 95%CI limit changing from a negative to a positive value which shows the signal first be found in 2013. From 2013 to 2018, the time scan map indicates a trend to a highly association and stable signal.

The time scans of HPV9-associated AEs are shown in Fig.3. In our research, only menstruation irregular was detected as signal by 2 methods and was a strong statistical association with the IC increasing, but the large 95% CI diminished possibility of a relationship between HPV9 and menstruation irregular. This signal was firt appeared in 2015. The combinations of HPV9-amenorrhea, FSH increased and premature menopause were not detected as a signal, and the time scans indicated a low possible association.

## Discussion

To our knowledge, this is the first signal detection research in which POI and related events were evaluated as a potential HPV vaccine adverse event. For the general characteristics of the reports, the reporter of healthcare providers accounted for 54.76% in HPV4, for 29.63% in HPV9, indicating that reports mainly accessed by professional staff. The patient age composition mainly concentrated on adolescent of 9–18 years old (HPV4 50.64%, HPV9 55.56%) and reproductive-age women between 19 and 26 years old (HPV4 24.94%, HPV9 14.81%), within the age for ovarian development and fertility. In addition, more than 70% of patients were younger than 40 years old, consistent with the epidemiological characteristics and diagnostic criteria of POI ^3^. SAE reports were few in this database, mainly in disability (6.43% for HPV4; 3.70% for HPV9). Although two methods, BCPNN and MGPS, were used to improve the signal detection, some positive signals might have been missed due to the low sensitivity of Bayesian method.

The American Society of Reproductive Medicine classified POI into occultation period, biochemical abnormality period and clinical abnormal period according to FSH level, menstrual status and fertility. The fertility is reduced with the increase of FSH as early manifestation, and finally develops into irregular menstruation or amenorrhea ^11^. Amenorrhea, FSH increased, and menstruation irregular are the symptoms and examination indicators of POI that serve as early warning on the development of POI for healthcare providers to identify POI early. Although POI is often used as synonyms of menopause, they are not equivalent. Thus, we conducted signal detection for amenorrhea, FSH increased, menstruation irregular, and premature menopause to assessing the sataistical risk of POI caused by HPV vaccines.

The signal detection results showed: (a) HPV2 did not yield any signal; (b) HPV4 was associated with amenorrhea, FSH increased, menstrual irregularities and premature menopause, indicating HPV4 with a sataistical risk of POI and related events; (c) irregular menstruation was detected as signal for HPV9 only. The diagrams of time scan showed: (a) because there was no signal for HPV2, no time scan figure was done for it; (b) the time scan of HPV4-associated POI related events showed that the signals were stable and strong association; (c) time scan of HPV9-POI related events indicated that the value of IC increasing but with a large 95% CI bars, suggesting the unstable signals. It was worth noting that the signals only represented a statistical relationship between HPV vaccines and adverse events, did not represent a causal relationship.

The possible pathogenesis between POI and HPV vaccines were multifactorial, such as autoimmune response prompted by HPV vaccines ^12^ and the adjuvant adverse effect of vaccine ^13^. A possible relationship between POI and HPV vaccination has been proposed in view of the temporal vaccine-event association ^12^. POI is a clinical disorder with complicated aetiology, and 5–30% of POI cases have an autoimmune mechanism ^14^. But the pathogenesis of HPV vaccine as a tigger factor for autoimmune disease is difficult to assess and did not found conclusive evidence ^12^. In immunology, according to defined by the National Cancer Institute, an adjuvant is an agent which might stimulate and increase the immune response to a vaccine, the aluminum salt was the most common adjuvant in human vaccines ^15^, and aluminum salt is the adjuvant in HPV2, HPV4 and HPV9. The adjuvant of aluminum salt could enhance or prolong antigen-specific immune response, but it may have potential adverse effect. According to animal experiments, aluminum could accumulate in the ovary, damage the structure of the ovary and decrease ovarian weight, which suppressed the concentrations of estrogen, progestogen, FSH, luteinizing hormone and resulted in abnormal development of ovarian follicles, and the reproductive function of female rats was inhibited by aluminum exposure ^16,17^.

Our study detected the signals of HPV4-associated POI related events and the signal of menstruation irregular for HPV9. Although our results were different from other reseauches, such as Naleway ^5^ and Pellegrino ^6^, which did not obversed an elevated risk of POI after HPV4 administration but they suspected presenting of a rare risk factor of determining the adverse event ^6^, our results provided a signal evidence in the view of pharmacovigilance. Furthermore, long-term reproductive safety studies are surely needed. HPV4 and HPV9 have the same preparation process and adjuvants except for the different viral subtypes. Short-term of marketed and lack of long-term safety data may be attribute to the difference in signals between the both. In addition, no studies have shown that the difference in adverse events due to different virus subtypes.

However, data mining and signal detection of VAERS has several limitations. First, the major limitations of our study are that it is unclear whether these AEs caused by disease, and lack of the data of medical history, medication history and menstrual history. Therefore, disease-oriented adverse events could be detected as signals. Second, VAERS is subject to various biased such as missing data, over-reporting, under-reporting. Third, because there is no specific value of FSH in VAERS and each report is only one FSH test result without continuous result, the FSH increase signal may bias to POI. Fourth, since combined vaccination may inhibit or enhance the immune response ^18^, this study only includes the reports of vaccination alone and excluding data from co-vaccination. But it resulted in a reduction in sample size and it is also a bias in our study. According to the clinical trials for HPV4 and HPV9 co-vaccination with other vaccines, they found that it is safety in vaccination combination, but lack of the data of post-marketed ^19,20^.

## Conclusions

AE reports in VAERS were reviewed to analysis the safety profiles of HPV vaccine-associated POI and related events. From the results of signal detection, it was suggesting that HPV4 have statistically signaificant association with POI, including amenorrhea, menstruation irregular, FSH increased and premature menopause, and HPV9 have potential statistical risk in menstruation irregular. However, it should be noted that our research data is accessed from VAERS and results are affected by the quantity and quality of AE reports, and studying POI as an HPV vaccine adverse event is challenging for many reasons. Finally, further research and cauaslity investigation between HPV vaccine and POI are strongly suggested in the future.

## Methods

### Data sources

VAERS is a passive surveillance system created by the Food and Drug Administration (FDA) and Centers for Disease Control and Prevention (CDC) to receive adverse event (AE) reports that may be possible associated with vaccines. The primary purpose of VAERS is to find early signal and generate hypotheses about possible new vaccine adverse events that do not be found during pre-market trails. VAERS is a free and open database, and VAERS data is accessible by two ways: downloading raw data in comma-separated value (CSV) files from https://vaers.hhs.gov/data/datasets.html, or using the CDC WONDER online search tool. The raw data in VAERS is presented by calendar year and updated monthly. The downloaded data include 3 separated data files, which are VAERSDATA, VAERSSYMTOMS, VAERSVAX, and each file have a unique ID for one report ^21^. In our study, we downloaded raw data from VAERS wepsite and extract data using ID.

In VAERSVAX file, HPV vaccine was encoded as HPVX, HPV2, HPV4, HPV9, so the search terms of this study were defined as HPV2, HPV4 and HPV9. The search time was set to the year of vaccine launch until 2018, with HPV2 from January 2009 to December 2018, HPV4 from January 2006 to December 2018, and HPV9 from January 2015 to December 2018. In order to avoid the impact of co-vaccination, this study only included adverse event reports of HPV vaccination alone. We used EXCEL 2016 to screen and extract data on target vaccine-adverse event reports, including: number of reports, age, the type of reporter, and serious adverse events.

### The definition of POI and related event

Medical Dictionary for Regulatory Activities (MedDRA) is a collection of international medical terminology prepared by the International Council for Harmonization (ICH) for the standardization and unification of adverse event report. MedDRA contains 5 hierarchical structures, which are Lowest Level Term (LLT), Preferred Term (PT), High Level Terms (HLT), High Group Terms (HLGT), System Organ Classes (SOC). Adverse events in VAERS reports are coded using the MedDRA of PT. We searched adverse events in MedDRA 19.0. Since MedDRA 19.0 did not include the term of POI, we redefined POI and related events according to definition and diagnostic criteria from ESHRE Guideline ^3^, which are oligomenorrhea/amenorrhea for at least 4 months, an elevated follicle stimulating hormone (FSH) level more than 25 IU/L on two occasion more than 4 weeks apart. We defined 3 related events: amenorrhea, irregular menstruation (including oligomenorrhea and delayed menstruation), and FSH increased to evaluate the association between HPV and POI. Considering that the two terms of POI and premature menopause are often used interchangeably ^5^, we also included the event of premature menopause into the study.

### Signal detection

The signals of disproportionality reports were detected by Bayesian methods, including Bayesian Confidence Propagation Neural Network (BCPNN)^22^ and Multi-item Gamma Poisson Shrinker (MGPS)^23,24^. The basis of those methods is based on two-by-two contingency table (Table 3).

**Table 3:** Two-by-two contingency table for disproportionality analysis.

	Reports with the target AE	All other AEs	Total
Reports with the target vaccine	a	b	a + b
All other vaccines	c	d	c + d
Total	a + c	b + d	a + b + c + d

The theory is to organize the reports of HPV-associated POI and related events in VAERS into the four analysis units of the vaccine-AE combination, including target vaccine-target AE, target vaccine-other AEs, other vaccine-target AE, other vaccines-other AEs. Then, we conducted statistical and disproportionalily analysis. In order to reduce the generation of false positive signals, our study used both BCPNN and MGPS methods to detect signals. When the results of both methods were positive, a signal was detected. The signal detection standards of each method are shown in Table 4.

**Table 4:** Signal detection standards of each method.

Method	Criterion
BCPNN^22^	a ≥ 3; IC-SD > 0
MGPS^23,24^	a ≥ 3; EB05 ≥ 2

In addition, this study will plot the time scan for HPV-associated POI and related events of information component(IC) and 95% CI, in order to reflect the change tendency of the target vaccine-target adverse event association in the database as the number of reports increasing over time, and verify the signal stability and association strength. When the IC curve is steady upward trend and the 95% CI narrowed, the signal is stable and strong association.

## Data Availability

The datasets generated and analysed during our study were available in the VAERS repository, https://vaers.hhs.gov/data/datasets.html.
